# Inducer-independent production of pectinases in *Aspergillus niger* by overexpression of the D-galacturonic acid-responsive transcription factor *gaaR*

**DOI:** 10.1007/s00253-018-8753-7

**Published:** 2018-01-24

**Authors:** Ebru Alazi, Tim Knetsch, Marcos Di Falco, Ian D. Reid, Mark Arentshorst, Jaap Visser, Adrian Tsang, Arthur F. J. Ram

**Affiliations:** 1 Molecular Microbiology and Biotechnology, Institute of Biology Leiden, Leiden University, Sylviusweg 72, 2333 BE Leiden, The Netherlands; 2 Centre for Structural and Functional Genomics, Concordia University, Québec, Canada

**Keywords:** Transcriptome, Exoproteome, Gene regulation, Transcription factor localization, GFP fluorescence, Transcription factor concentration

## Abstract

The transcription factor GaaR is needed for the expression of genes required for pectin degradation and transport and catabolism of the main degradation product, D-galacturonic acid (GA) in *Aspergillus niger*. In this study, we used the strong constitutive *gpdA* promoter of *Aspergillus nidulans* to overexpress *gaaR* in *A. niger*. Overexpression of *gaaR* resulted in an increased transcription of the genes encoding pectinases, (putative) GA transporters, and catabolic pathway enzymes even under non-inducing conditions, i.e., in the absence of GA. Exoproteome analysis of a strain overexpressing *gaaR* showed that this strain secretes highly elevated levels of pectinases when grown in fructose. The genes encoding exo-polygalacturonases were found to be subjected to CreA-mediated carbon catabolite repression, even in the presence of fructose. Deletion of *creA* in the strain overexpressing *gaaR* resulted in a further increase in pectinase production in fructose. We showed that GaaR localizes mainly in the nucleus regardless of the presence of an inducer, and that overexpression of *gaaR* leads to an increased concentration of GaaR in the nucleus.

**Electronic supplementary material:**

The online version of this article (10.1007/s00253-018-8753-7) contains supplementary material, which is available to authorized users.

## Introduction

*Aspergillus niger* is an important filamentous fungus for the industrial production of pectinases (Pedrolli et al. 2009). Pectinases are widely used in the food industry (Kashyap et al. 2001; Toushik et al. 2017; Khan et al. 2013) and are important enzymes in the utilization of pectin-rich feedstock in biofuel production (Edwards and Doran-Peterson 2012). Pectin is a complex plant cell wall polysaccharide and four substructures have been defined which include polygalacturonic acid (PGA), rhamnogalacturonan I, rhamnogalacturonan II, and xylogalacturonan. PGA is the most abundant pectic substructure and consists of D-galacturonic acid (GA) residues. GA is also present in the backbones of rhamnogalacturonan I, rhamnogalacturonan II, and xylogalacturonan (Caffall and Mohnen 2009).

*A. niger* contains a large number of enzymes potentially acting on pectin substructures (Martens-Uzunova and Schaap 2009; Coutinho et al. 2009; De Vries et al. 2017). In the presence of GA, the main sugar acid in pectin, the expression of the genes encoding pectinases (Martens-Uzunova and Schaap 2009; Alazi et al. 2016), the GA transporter GatA (Sloothaak et al. 2014), and the GA catabolic pathway enzymes GaaA, GaaB, GaaC, and GaaD (Martens-Uzunova and Schaap 2008; Alazi et al. 2016) are induced via the Zn_2_Cys_6_ type transcription factor GaaR (Alazi et al. 2016). Apart from the transcriptional activator (GaaR), the expression of GA-responsive genes is controlled by a repressor protein, GaaX. Loss of function of GaaX leads to constitutive and inducer-independent expression of pectinases (Niu et al. 2017). The repressor protein GaaX is postulated to inhibit the transcriptional activity of GaaR under non-inducing conditions, i.e., in the absence of GA. The presence of an inducer is suggested to inhibit the repressing activity of GaaX, thereby leading to the transcriptional induction of GA-responsive genes via GaaR (Niu et al. 2017). The GA catabolic pathway intermediate 2-keto-3-deoxy-L-galactonate has recently been identified as the physiological inducer of the GA-responsive genes (Alazi et al. 2017).

Overexpression of transcription factors has been shown to be an effective method to increase the expression of their target genes in *Saccharomyces cerevisiae*, even under conditions in which the transcription factors under consideration are normally not active (Chua et al. 2006). Similarly, overexpression of transcription factors involved in plant biomass degradation in filamentous fungi, such as *xlnR* (Noguchi et al. 2009) and *manR* (Ogawa et al. 2012) in *Aspergillus oryzae* and *xyr1* in *Trichoderma reesei* (Jiang et al. 2016), was previously reported to result in elevated expression of their target genes in the presence of inducers. Inducer-independent production of cellulases was also observed in *T. reesei* strains overexpressing *xyr1* (Lv et al. 2015; Wang et al. 2013).

In this study, we demonstrate that overexpression of *gaaR* results in constitutive transcription and secretion of pectinases under non-inducing conditions, probably by disturbing the stoichiometric balance of GaaR and GaaX in favor of GaaR. We further show that the effect of *gaaR* overexpression on pectinase production is sensitive to CreA-mediated carbon catabolite repression even when fructose, a less repressing carbon source compared to glucose, was used. A further increase in pectinase production on fructose upon *gaaR* overexpression was accomplished when the CreA-mediated carbon catabolite repression was inactivated via *creA* deletion.

## Materials and methods

### Strains, media, and growth conditions

All *A. niger* strains used in this study are listed in Online Resource 1. Media were prepared as described previously (Arentshorst et al. 2012). Radial growth assays of the strains were performed on minimal medium (MM) (pH 5.8) containing 1.5% (*w*/*v*) agar (Scharlau, Barcelona, Spain) and various carbon sources: 50 mM glucose (VWR International, Amsterdam, The Netherlands), fructose (Sigma-Aldrich, Zwijndrecht, The Netherlands), or GA (Chemodex, St Gallen, Switzerland), or 1% (*w*/*v*) PGA (Sigma, Zwijndrecht, The Netherlands) or apple pectin (AP) (Sigma-Aldrich, Zwijndrecht, The Netherlands). Plates were inoculated with 5 μL 0.9% NaCl containing 10^4^ freshly harvested spores and cultivated at 30 °C for 7 days. MM (pH 5.8) containing 1.5% (*w*/*v*) agar, 10 mM acetamide (Sigma-Aldrich, Steinheim, Germany) as the sole nitrogen source, and acetate (Merck, Darmstadt, Germany), glucose, fructose, sorbitol (Roth, Karlsruhe, Germany) or GA as the carbon source was prepared as described previously (Arentshorst et al. 2012). Plates were inoculated with 5 μL 0.9% NaCl containing 5 × 10^4^ freshly harvested spores. Filter sterilized carbon source solutions were added after autoclaving MM containing agar. PGA and AP were autoclaved together with the medium. All growth experiments were performed in duplicate.

For enzymatic analysis, 10^6^ freshly harvested spores were inoculated per mL in 100 mL shake flasks that include 25 or 50 mL MM (pH 5.8) containing 50 mM glucose, fructose, sorbitol, or GA and were grown for 36 h in a rotary shaker at 30 °C and 250 rpm. Experiments were performed in duplicate.

For microscopic analysis of the co-localization of the nuclear specific SYTO59 dye (Invitrogen, Eugene, Oregon, USA) with the eGFP-tagged H2B protein, conidia of the MA26.1 strain were propagated on complete medium containing 1.5% (*w*/*v*) agar. 2 × 10^5^ freshly harvested spores were placed on cover slips in a Petri dish with 20 mL MM containing 50 mM fructose and grown at 30 °C. After 16 h, the cover slips were rinsed twice with water and transferred to a new Petri dish with 20 mL MM containing 50 mM GA and growth was continued at 30 °C for 1.5 h. For microscopic analysis of the co-localization of the nuclear specific SYTO59 dye with the eGFP-tagged GaaX or GaaR proteins, conidia of the JN126.2, EA19.2, and EA20.10 strains were propagated on MM containing 1.5% (*w*/*v*) agar and 50 mM GA. 3 × 10^5^ freshly harvested spores were inoculated on cover slips in Petri dishes that include 3 mL MM containing10 mM GA and 0.003% yeast extract and grown at 30 °C for approximately 22 h. For microscopic analysis of the fluorescence intensity, conidia of the EA19.2 and EA20.10 strains were propagated on complete medium containing 1.5% (*w*/*v*) agar. 2 × 10^5^ freshly harvested spores were placed on cover slips in Petri dishes that include 20 mL MM containing 50 mM fructose, or 50 mM GA and 1 mM fructose, and grown at 30 °C for 17.5 h. For each condition, two biological replicates were performed.

### Construction of strains overexpressing *gaaR*

Protoplast-mediated transformation of *A. niger*, purification of the transformants and extraction of the genomic DNA were performed as described by Arentshorst et al. (2012).

The plasmid pEA4 containing the P*gpdA*-*gaaR*-T*trpC* construct was created as follows: the *Aspergillus nidulans gpdA* promoter was obtained from plasmid pAN52.1-NOTI (Punt et al. 1987) by restriction digestion with *Not*I and *Nco*I. The *gaaR* gene was amplified by PCR using the primer pairs listed in Online Resource 2 with *A. niger* N402 genomic DNA as template, ligated into pJET1.2/blunt cloning vector (Thermo Fisher Scientific, Carlsbad, CA) and amplified in *Escherichia coli* DH5*α*. Following plasmid isolation, *gaaR* was excised using restriction enzymes *Psc*I and *Bgl*II. The *Not*I-*Nco*I fragment of P*gpdA* and the *Psc*I-*Bgl*II fragment of *gaaR* were ligated into *Not*I-*Bam*HI opened pAN52.1-NOTI. pEA4 was sequenced to ensure no PCR errors have occurred and proper ligation and orientation of the fragments. To create strains EA21.3, EA21.5, EA21.6, and EA21.8, pEA4 was co-transformed into strain JN36.1 together with the plasmid pMA357 containing the *A. nidulans amdS* gene behind the *A. nidulans gpdA* promoter (Alazi et al. 2016). Transformants were selected on plates containing acetamide as the sole nitrogen source. To create strain TK1.1, strain JN36.1 was co-transformed with pEA4 and the plasmid p3SR2 (Hynes et al. 1983). p3SR2 contains the *A. nidulans amdS* gene behind the endogenous *amdS* promoter (Hynes et al. 1983). Transformants were selected on plates containing acrylamide as the sole nitrogen source. Ectopic integration of the P*gpdA*-*gaaR*-T*trpC* construct was confirmed via Southern blot analysis. Genomic DNA was restricted overnight with *Nco*I restriction enzyme. A 501 bp fragment containing the *gaaR* gene was PCR-amplified using the primer pairs listed in Online Resource 2 with N402 genomic DNA as template, and was used as a probe.

### Construction of strains (over)expressing *eGFP-gaaR*

The *gaaR* and *eGFP* genes were amplified by PCR using the primer pairs listed in Online Resource 2 with N402 genomic DNA and the plasmid pFG029 (unpublished vector, containing P*gpdA*-*eGFP*-T*trpC*) as template, respectively. *eGFP* and *gaaR* were combined by fusion PCR using primers eGFP_P1_NcoI and gaaR_comp_P2_BglII, and the *eGFP*-*gaaR* fusion product was ligated into pJET1.2/blunt cloning vector and amplified in *E. coli* DH5*α*. Following plasmid isolation, the *eGFP*-*gaaR* fusion product was excised in two parts using restriction enzymes *Nco*I and *Bgl*II, resulting in an *Nco*I-*Nco*I fragment and an *Nco*I-*Bgl*II fragment.

The plasmid pEA3 containing the P*gpdA-eGFP-gaaR*-T*trpC* construct was created as follows: The *Not*I-*Nco*I fragment of P*gpdA* and the *Nco*I-*Bgl*II fragment of *eGFP*-*gaaR* were ligated into *Not*I-*Bam*HI opened pAN52.1-NOTI. The resulting plasmid was digested with *Nco*I and ligated with the *Nco*I-*Nco*I fragment of *eGFP*-*gaaR*. pEA3 was sequenced to ensure no PCR errors and proper ligation and orientation of the fragments. Strain EA20.10 was created by co-transformation of strain JN36.1 with pEA3 together with the plasmid pMA357.

To construct plasmid pEA2 (P*gaaR-eGFP-gaaR*-T*trpC)*, the *gaaR* promoter was PCR-amplified using the primer pairs listed in Online Resource 2 with N402 genomic DNA as template, ligated into pJET1.2/blunt cloning vector and amplified in *E. coli* DH5*α*. Following plasmid isolation, P*gaaR* was excised using restriction enzymes *Not*I and *Nco*I. pEA2 was created in a similar way to pEA3, except that the *Not*I-*Nco*I fragment of P*gaaR* was used instead of P*gpdA*. pEA2 was sequenced to ensure no PCR errors and proper ligation and orientation of the fragments. Strain EA19.2 was created by co-transformation of strain JN36.1 with pEA2 together with the plasmid pMA357 and transformants were selected on plates containing acetamide as the sole nitrogen source. Ectopic integrations of the P*gpdA-eGFP-gaaR*-T*trpC* and P*gaaR-eGFP-gaaR*-T*trpC* constructs were confirmed by diagnostic PCRs (data not shown).

### Construction of *creA* deletion strains

Loss of the *pyrE* gene in EA21.6 was mediated by counter selection on MM-5’-FOA plates (Arentshorst et al. 2012), resulting in the strain EA23.6. The split marker approach was employed in the deletion of the *creA *gene (Arentshorst et al. 2015). 5′ and 3′ flanks of *creA* were PCR-amplified using the primer pairs listed in Online Resource 2 with N402 genomic DNA as template. The *A. nidulans pyrF* gene (named *pyrE* in *A. niger*) was PCR-amplified as two fragments using the primer pairs listed in Online Resource 2 with *A. nidulans* strain A234genomic DNA as template. Split marker fragments with the *pyrF* selection marker were created by fusion PCR and used to transform the strain EA23.6, resulting in the strain TK2.1. Proper deletion of *creA* was confirmed by diagnostic PCR (data not shown).

MA342.2 was also constructed using the split marker approach (Arentshorst et al. 2015). 5′ and 3′ flanks of *creA* were PCR-amplified using the primer pairs listed in Online Resource 2 with N402 genomic DNA as template. The hygromycin resistance cassette was PCR-amplified using primers hygP3f and hygP4r and a derivative of pAN7.1 (Punt et al. 1987) as template. *creA-hygR* split marker fragments were created by fusion PCR and transformed to strain MA234.1, resulting in the *ΔcreA* strain MA342.2. Proper deletion of *creA* was confirmed by diagnostic PCR (data not shown).

### Bioreactor cultivations and transcriptome and exoproteome analyses

Controlled bioreactor cultivations of MA234.1 (the reference strain) (in triplicate) and JN123.1 (*ΔgaaX*) (in duplicate) in MM containing 0.75% fructose and the subsequent transcriptome analyses were performed previously (Niu et al. 2017). Controlled bioreactor cultivations of the EA21.6strain (*OEgaaR*) (in duplicate) under exactly the same growth conditions and the subsequent RNA-seq analyses were performed as previously described by Niu et al. (2017). Both biomass accumulation (offline) and base addition (online) were determined to monitor exponential growth.

Broth samples were taken during exponential growth after every 4 mL of base addition. RNA isolated from exponentially growing cells at the sample point at which about 75–80% of the maximum biomass yield was reached was used for the RNA-seq experiment. RNA-seq data were submitted to the Sequence Read Archive under accession number SRP078485 for MA234.1 and JN123.1 (Niu et al. 2017) and accession number SRP114830 for EA21.6 (this study).

Supernatant samples from an exponentially growing culture of each strain at two successive sample points (based on base addition) following the RNA-seq sample point were withdrawn and filtered. The filtered supernatants were lyophilized, resuspended in 1 mL 50 mM citric acid buffer pH 5.0, and used for the exoproteome analysis. For each sample, proteins were precipitated with TCA (trichloroacetic acid), the pellet was washed twice with acetone and resuspended in 75 μL 200 mM ammonium bicarbonate and 0.1% AALS II. Protein concentrations were determined by the RCDC assay Kit (BioRad, Mississauga, Ontario). Three micrograms of total protein were loaded on 12% SDS-PAGE gel. The gel was colored with silver stain and developed for 3 min. Five micrograms of total protein were trypsin digested in solution overnight at 37 °C. Samples were desalted with C18 ziptips (Millipore, Billerica, MA), the eluate was dried and the peptides were resuspended in 50 μL 5% acetonitrile and 0.1% formic acid. Five microlitres of peptide digest were analyzed by LC-MS/MS on a Velos LTQ-Orbitrap. Extracted Ion Chromatogram (EIC) peak area values of proteins were calculated by averaging the top three most abundant peptide ion EIC values assigned to each protein as per the Proteome Discoverer 1.4 (Thermo Fisher, San Jose, CA) precursor peak area quantification workflow. Protein EIC area values were normalized using the determined value of a fixed spiked amount of trypsin-digested bovine serum albumin.

### Enzymatic analysis

Supernatants from bioreactor or shake flask cultures were obtained by filtration through glass microfiber filters (Whatman, Buckinghamshire, UK) or sterile miracloth, and the filtrate was stored at − 80 °C. PGA plate assays were performed as described by Niu et al. (2017). Twenty-five microlitres of supernatant from each culture was spotted on plates containing 0.2% or 0.5% PGA, and plates were incubated at 37 °C for 16 or 20 h. PGA degradation was indicated by the formation of a clear zone of hydrolysis.

### Microscopy

The coverslips with adherent germlings were placed upside down on glass slides and observed under a Zeiss Observer confocal laser scanning microscope (Zeiss, Jena, Germany). For nuclear staining, 0.5 mL of 25 μM SYTO59 dye solution was dropped on glass slides before placing the cover slips, and imaging was performed approximately after 2 h. The GFP and SYTO59 fluorescence were excited using 488 and 625 nm laser lines, respectively. Images were analyzed using the ImageJ software (Abramoff et al. 2004). To analyze the fluorescence intensity, 1–2 images were taken for each biological replicate. On each image, the exact same brightness and contrast adjustments were applied, 3–10 nuclear and 3–10 cytoplasmic fluorescence intensities in a defined area were measured, and calibrated for the background fluorescence.

## Results

### Expression of pectinase genes is upregulated in strains overexpressing *gaaR*

To create strains that overexpress the GA-responsive transcription factor gene *gaaR* (*OEgaaR*), the *A. niger gaaR* gene was fused with the strong constitutive *A. nidulans gpdA* promoter and transformed into a *ΔgaaR* strain. Southern blot analysis indicated that the *gaaR* overexpression construct was ectopically integrated in one or two copies in the genomes of the *OEgaaR* strains EA21.3, EA21.5, EA21.6, and EA21.8 (Fig. ESM_3.1 b). An additional multicopy *OEgaaR* strain, TK1.1, was obtained by using a more stringent selection method using acrylamide, and Southern blot analysis confirmed ectopic integration of at least four copies of the *gaaR* overexpression construct in its genome (Fig. ESM_3.1 c). We compared the radial growth of the *OEgaaR* strains on different monomeric and polymeric carbon sources (Fig. ESM_3.2). The *ΔgaaR* strain showed a strongly reduced growth on GA, PGA, and AP as previously shown (Alazi et al. 2016). Reintroducing the *gaaR* gene expressed from the *gpdA* promoter resulted in growth on GA, PGA, and AP in EA21.3, EA21.5, EA21.6, and EA21.8, indicating the presence of a functional GaaR. However, the *OEgaaR* strains showed partial and different levels of complementation of growth on GA-containing carbon sources (Fig. ESM_3.2 a). The TK1.1 strain, containing the highest copy number of the *gaaR* overexpression construct, showed a severely impaired growth on GA, PGA, and AP (data not shown and Fig. ESM_3.2 b). Growth of all *OEgaaR* strains on glucose or fructose was similar to the growth of the reference strain.

To assess the pectinase production capacity of the *OEgaaR* strains, the strains were grown in shake flasks in minimal medium containing non-inducing (50 mM glucose, 50 mM fructose, or 50 mM sorbitol) or inducing (50 mMGA) carbon sources, and the culture supernatants were spotted on PGA plates. As indicated by the clear zones of hydrolysis on PGA plates, the polygalacturonase activity in the culture supernatants of the *OEgaaR* strains EA21.3, EA21.5, EA21.6, and EA21.8 grown in glucose or fructose was higher compared to the reference strain (Fig. ESM_3.3 a). The culture supernatant of EA21.6 grown on sorbitol or GA displayed the highest polygalacturonase activity compared to EA21.3, EA21.5, EA21.8, and the reference strain (Fig. ESM_3.3 a and data not shown). EA21.6 was selected to be used in further experiments based on the growth profiles and pectinase production capacities of the *OEgaaR* strains. A further increased level of polygalacturonase activity was observed in the culture supernatant of TK1.1 grown on fructose compared to the reference strain as well as to EA21.6 (Fig. ESM_3.3 b).

### Transcriptome analysis of the *OEgaaR* strain

**Fig. 1:**
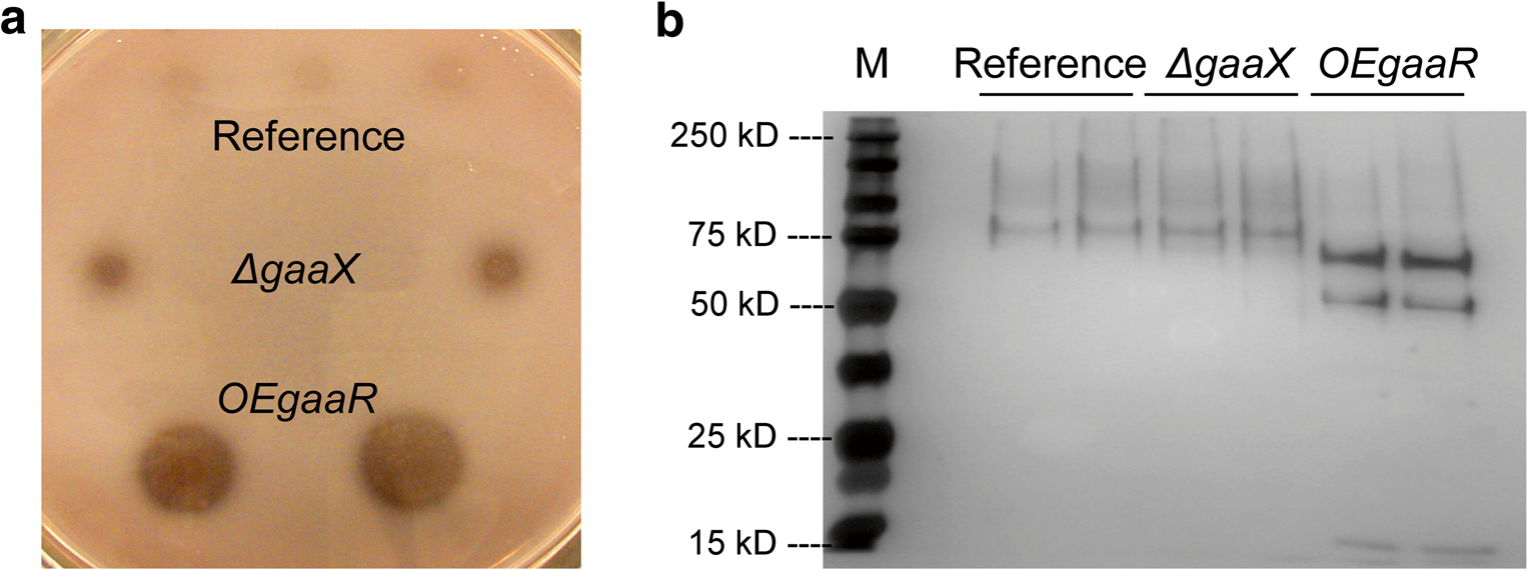
Enzymatic analysis and secretome profiles of the *A. niger* reference (MA234.1), *∆gaaX* (JN123.1) and *OEgaaR* (EA21.6) strains grown in bioreactors on 0.75% fructose. **a** PGA plate assay. Supernatant from each bioreactor culture at the sample point following the RNA-seq sample point was spotted on a PGA plate. **b** Silver stained SDS-PAGE patterns of secretomes from a bioreactor culture of each strain at two successive sample points following the RNA-seq sample point. Three micrograms of total protein were loaded in each lane. Marker (M) molecular weight in kD is indicated

To investigate the expression of the multitude of genes involved in pectin degradation, GA transport, and catabolism in a strain overexpressing *gaaR*, we performed a genome-wide gene expression analysis using RNA-seq (Online Resource 4). The reference and *OEgaaR* (EA21.6) strains were grown in bioreactors on fructose, a carbon source that does not induce the expression of GA-responsive genes (Martens-Uzunova and Schaap 2008). Growth of the *OEgaaR* strain under the controlled bioreactor conditions (μ_max_ 0.200 ± 0.001 g dry weight/kg/h, *Y*_max_ 4.117 ± 0.167 g dry weight/kg (*n* = 2)) was similar to the growth of the reference strain (*μ*_max_ 0.214 ± 0.007 g dry weight/kg/h, *Y*_ma*x*_ 4.151 ± 0.134 g dry weight/kg (*n* = 3)). Analysis of RNA-seq data showed that the expression of *gaaR* was highly increased in the *OEgaaR* strain compared to the reference strain with a fold change of 63.8 (Online Resource 4 and Table 1). Overexpression of *gaaR* resulted in the upregulation (FC ≥ 4, FDR ≤ 0.001) of 19 of 48 genes encoding extracellular enzymes specifically assigned to the degradation of pectin according to de Vries et al. (2017) (see Table 1). Almost all of these genes (18 out of 19) belong to the GaaR/GaaX panregulon (Niu et al. 2017) and include several exo- and endo-polygalacturonases, pectin methylesterases, and pectin lyases, all acting on the PGA backbone of pectin, as well as the xylogalacturonase NRRL3_07469 acting on xylogalacturonan, and the arabinogalactanase encoded by *gan53A* and the *α*-L-rhamnosidase NRRL3_10558 acting on rhamnogalacturonan I. Nine of 19 pectinases that were upregulated in the *OEgaaR* strain were previously shown to be upregulated in *ΔgaaX* (JN123.1), the repressor deletion mutant, on fructose compared to the reference strain (FC ≥ 4, FDR ≤ 0.001) (Niu et al. 2017). The expression of these nine pectinase genes, except NRRL3_05252, was generally much lower in *ΔgaaX* than in the *OEgaaR* strain, and no additional pectinases were found to be upregulated in *ΔgaaX* (Table 1). Constitutive production of pectinases in the *OEgaaR* strain and to a lesser extent in *ΔgaaX* grown in bioreactors on fructose was also observed via a PGA plate assay (Fig. 1a). This analysis clearly indicates that overexpression of *gaaR* results in a more dramatic increase in the expression of several pectinases compared to deletion of the repressor *gaaX*.

**Table 1:** Transcriptome and exoproteome analyses of predictive pectinases, GA transporters and catabolic pathway genes, *gaaR* and *gaaX* in the reference, *OEgaaR* and *ΔgaaX* strains. Transcript and extracellular protein levels are represented as TPM and normalized protein (EIC) area values, respectively. Transcript levels with a fold change≥4 and FDR≤0.001 compared to the reference strain are indicated with an asterisk. Genes belonging to the GaaR/GaaX panregulon (Niu et al. 2017) are written in bold. Transcriptome data for the reference and *ΔgaaX* strains were taken from Niu et al. 2017

**Gene ID NRRL3**	**Gene ID** **CBS 513.88**	**Description**	**CAZy family**	**Transcript level (ave TPM)**			**Extracellular protein level** **(ave normalized protein (EIC) area)**		
				**Reference**	***ΔgaaX***	***OEgaaR***	**Reference**	***ΔgaaX***	***OEgaaR***
NRRL3_00169	An09g02160	rhamnogalacturonan acetylesterase	CE12	0.455	0.680	0.822	#N/A	#N/A	#N/A
NRRL3_07501	An04g09360	carbohydrate esterase family 12 protein	CE12	1.313	1.134	1.680	0.00	0.00	1.31
**NRRL3_08325**	**An03g06310**	**pectin methylesterase Pme8A**	CE8	0.037	0.556*	24.737*	0.00	1.48	105.91
**NRRL3_07470**	**An04g09690**	**pectin methylesterase**	CE8	0.755	5.722*	57.977*	0.55	7.49	45.73
**NRRL3_05252**	**An02g12505**	**pectin methylesterase-like protein**	CE8	1.061	31.320*	17.513*	0.00	109.39	48.98
NRRL3_00839	An14g02920	glycoside hydrolase family 105 protein	GH105	4.997	5.090	4.027	#N/A	#N/A	#N/A
NRRL3_01038	An14g05340	glycoside hydrolase family 105 protein	GH105	0.330	0.367	0.289	#N/A	#N/A	#N/A
NRRL3_06782	An16g06990	endo-polygalacturonase Pga28A	GH28	0.000	0.000	0.000	8.56	11.51	19.74
NRRL3_05859	An02g04900	endo-polygalacturonase Pga28B	GH28	16.541	18.675	27.407	0.00	0.22	1.76
**NRRL3_08805**	**An05g02440**	**endo-polygalacturonase Pga28C**	GH28	0.053	0.596	376.634*	0.00	0.00	321.96
NRRL3_00263	An09g03260	endo-polygalacturonase Pga28D	GH28	0.929	1.066	1.322	0.605	0	0
**NRRL3_02835**	**An01g14670**	**endo-polygalacturonase Pga28E**	GH28	0.483	1.152	479.334*	0.00	0.00	415.78
**NRRL3_02571**	**An01g11520**	**endo-polygalacturonase Pga28I**	GH28	0.215	1.243	23.092*	0.00	0.00	8.40
NRRL3_04000	An15g05370	endo-polygalacturonase Pga28II	GH28	0.293	1.055	2.076*	0.00	2.94	11.24
**NRRL3_03144**	**An12g07500**	**exo-polygalacturonase**	GH28	1.361	51.633*	65.839*	0.00	23.17	46.86
**NRRL3_08281**	**An03g06740**	**exo-polygalacturonase Pgx28B**	GH28	0.000	2.102*	14.290*	0.00	1.04	7.85
**NRRL3_05260**	**An02g12450**	**exo-polygalacturonase Pgx28C**	GH28	0.955	16.768*	45.050*	0.00	10.00	27.24
**NRRL3_09810**	**An11g04040**	**exo-polygalacturonase**	GH28	0.007	0.120	1.221*	#N/A	#N/A	#N/A
NRRL3_07469	An04g09700	xylogalacturonase	GH28	0.167	0.344	4.828*	0.00	0.00	10.86
NRRL3_09126	An12g00950	endo-rhamnogalacturonase Rhg28A	GH28	5.967	6.717	4.859	#N/A	#N/A	#N/A
NRRL3_00953	An14g04200	endo-rhamnogalacturonase Rhg28B	GH28	0.000	0.009	0.000	0.69	2.31	0.00
NRRL3_04303	An07g01000	endo-rhamnogalacturonase	GH28	0.183	0.249	0.256	#N/A	#N/A	#N/A
NRRL3_09450	An11g08700	endo-rhamnogalacturonase	GH28	1.380	1.585	1.286	#N/A	#N/A	#N/A
NRRL3_11790	An06g02070	endo-rhamnogalacturonase	GH28	0.217	0.061	0.034	#N/A	#N/A	#N/A
NRRL3_09623	An11g06320	endo-rhamnogalacturonase	GH28	0.169	0.301	0.035	#N/A	#N/A	#N/A
NRRL3_02832	An01g14650	glycoside hydrolase family 28 protein	GH28	3.472	5.049	3.482	#N/A	#N/A	#N/A
NRRL3_08631	An03g02080	alpha-L-rhamnosidase Rha28B	GH28	0.000	0.021	0.159	#N/A	#N/A	#N/A
**NRRL3_10559**	**An18g04810**	**glycoside hydrolase family 28 protein**	GH28	0.084	3.112*	16.880*	0.00	2.34	7.39
**NRRL3_10643**	**An18g05940**	**arabinogalactanase Gan53A**	GH53	1.738	3.850	28.517*	0.00	2.91	22.91
NRRL3_02162	An01g06620	alpha-L-rhamnosidase	GH78	9.524	8.130	8.251	#N/A	#N/A	#N/A
NRRL3_03279	An12g05700	alpha-L-rhamnosidase-like protein	GH78	1.326	1.329	0.921	#N/A	#N/A	#N/A
NRRL3_03924	An15g04530	glycoside hydrolase family 78 protein	GH78	0.000	0.000	0.000	#N/A	#N/A	#N/A
NRRL3_04245	An07g00240	alpha-L-rhamnosidase-like protein	GH78	0.070	0.077	0.043	#N/A	#N/A	#N/A
NRRL3_06304	An10g00290	alpha-L-rhamnosidase-like protein	GH78	1.072	1.365	1.259	#N/A	#N/A	#N/A
NRRL3_07520	An04g09070	alpha-L-rhamnosidase	GH78	2.228	2.178	1.749	#N/A	#N/A	#N/A
NRRL3_11451	An08g09140	alpha-L-rhamnosidase-like protein	GH78	0.085	0.166	0.009	#N/A	#N/A	#N/A
**NRRL3_10558**	**An18g04800**	**alpha-L-rhamnosidase**	GH78	0.350	3.847*	17.470*	#N/A	#N/A	#N/A
NRRL3_01739	An01g01340	glycoside hydrolase family 88 protein	GH88	0.163	0.219	0.212	#N/A	#N/A	#N/A
NRRL3_00824		sialidase-like protein	GH93	149.296	144.208	132.943	#N/A	#N/A	#N/A
**NRRL3_00965**	**An14g04370**	**pectin lyase Pel1A**	PL1_4	1.657	3.255	332.933*	2.21	2.57	653.22
NRRL3_08767	An03g00190	pectin lyase Pel1B	PL1_4	5.072	5.031	2.944	#N/A	#N/A	#N/A
NRRL3_04153	An15g07160	pectin lyase Pel1C	PL1_4	18.220	18.640	18.246	#N/A	#N/A	#N/A
NRRL3_06269		pectin lyase	PL1_4	0.015	0.033	0.083	#N/A	#N/A	#N/A
**NRRL3_01237**	**An19g00270**	**pectin lyase**	PL1_4	0.174	1.475*	13.613*	0.00	0.00	20.51
**NRRL3_09811**	**An11g04030**	**pectin lyase**	PL1_4	0.000	0.011	0.503*	#N/A	#N/A	#N/A
NRRL3_06359	An10g00870	pectate lyase Ply1A	PL1_7	0.148	0.134	1.366	#N/A	#N/A	#N/A
NRRL3_00684	An14g01130	rhamnogalacturonan lyase	PL4_1	0.021	0.052	0.320	#N/A	#N/A	#N/A
**NRRL3_10115**	**An11g00390**	**rhamnogalacturonan lyase**	PL4_3	0.648	0.810	11.176	#N/A	#N/A	#N/A
**NRRL3_00958**	**An14g04280**	**MFS-type sugar/inositol transporter GatA**		3.472	524.952*	125.820*	#N/A	#N/A	#N/A
**NRRL3_08663**	**An03g01620**	**MFS-type sugar/inositol transporter**		0.274	5.622*	0.577	#N/A	#N/A	#N/A
**NRRL3_04281**	**An07g00780**	**MFS-type transporter**		3.110	4.596	13.958*	#N/A	#N/A	#N/A
**NRRL3_05650**	**An02g07710**	**D-galacturonate reductase GaaA**		19.917	1515.440*	135.009*	#N/A	#N/A	#N/A
**NRRL3_06890**	**An16g05390**	**L-galactonate dehydratase GaaB**		47.765	6256.695*	782.361*	#N/A	3.78	#N/A
**NRRL3_05649**	**An02g07720**	**2-keto-3-deoxy-L-galactonate aldolase GaaC**		12.536	2283.765*	240.798*	#N/A	2.23	#N/A
**NRRL3_10050**	**An11g01120**	**L-glyceraldehyde reductase GaaD**		256.409	2732.370*	570.508	#N/A	6.00	0.24
NRRL3_08195	An04g00780	D-galacturonic acid responsive transcription factor GaaR		14.444	18.512	850.848*	#N/A	#N/A	#N/A
**NRRL3_08194**	**An04g00790**	**Repressor of D-galacturonic acid utilization GaaX**		15.968	0.000	352.560*	#N/A	#N/A	#N/A

Comparison of the expression of the genes encoding the (putative) GA transporters and the GA catabolic pathway enzymes (*gaaA*, *gaaB*, *gaaC*, and *gaaD*) between the *OEgaaR*, the reference and *ΔgaaX* strains revealed that *gatA*, the putative GA transporter NRRL3_04281, *gaaA*, *gaaB*, and *gaaC* were significantly upregulated (FC ≥ 4, FDR ≤ 0.001) in the *OEgaaR* strain (Table 1). The expression of *gaaD* was also significantly (FDR ≤ 0.001) increased in the *OEgaaR* strain compared to the reference strain with a fold change of 2.5 (Table 1). Interestingly, the expression of the genes encoding the GA catabolic pathway enzymes were moderately induced in the *OEgaaR* strain and expressed at much higher levels in the *ΔgaaX* strain. In contrast, many of the genes encoding the extracellular enzymes were expressed at higher levels in the *OEgaaR* strain compared to the *ΔgaaX* strain (see “Discussion”).

We also analyzed the effect of overexpression of *gaaR* on the expression of all 375 genes predicted to encode carbohydrate active enzymes (CAZymes) in *A. niger* strain NRRL3 (Online Resource 4). In addition to the above mentioned 19 pectinases (belonging to CAZy families CE8, GH28, GH53, GH78, PL1_4, and PL4_3), 20 CAZymes acting specifically on cellulose (AA9, GH5_5), starch (GH13_5, GH31), and xyloglucan (GH12, GH74, GH95) or acting on multiple substrates (CE16, GH18, GH3, GH35, GH43, GH51, GH54, GH79) (de Vries et al. 2017) were highly upregulated in the *OEgaaR* strain on fructose (Table 2).

**Table 2:** Transcriptome and exoproteome analyses of 20 additional CAZymes upregulated in the *OEgaaR* strain compared to the reference strain. Transcript and extracellular protein levels are represented as TPM and normalized protein (EIC) area values, respectively. Transcript levels with a fold change≥4 and FDR≤0.001 compared to the reference strain are indicated with an asterisk. Genes belonging to the GaaR/GaaX panregulon (Niu et al. 2017) are written in bold. Transcriptome data for the reference and *ΔgaaX* strains were taken from Niu et al. 2017

**Gene ID NRRL3**	**Gene ID** **CBS 513.88**	**Description**	**CAZy family**	**Transcript level (ave TPM)**			**Extracellular protein level** **(ave normalized protein (EIC) area)**		
				**Reference**	***ΔgaaX***	***OEgaaR***	**Reference**	***ΔgaaX***	***OEgaaR***
NRRL3_00814	An14g02670	lytic polysaccharide monooxygenase	AA9	1.053	2.625	20.043*	#N/A	#N/A	#N/A
NRRL3_06379		acetylesterase	CE16	0.042	0.142	24.511*	0.00	0.00	0.84
**NRRL3_06053**	**An02g02540**	**carbohydrate esterase family 16 protein**	CE16	2.055	17.617*	105.698*	0.00	12.71	71.49
NRRL3_01918	An01g03340	xyloglucanase Xeg12A	GH12	9.203	11.484	911.087*	#N/A	#N/A	#N/A
NRRL3_02746	An01g13610	glucan 1,4-alpha-maltohexaosidase	GH13_5	2.813	5.088	15.211*	#N/A	#N/A	#N/A
NRRL3_01212		chitinase-like protein	GH18	8.261	25.549	43.967*	0.00	0.00	0.34
NRRL3_06419	An17g00300	glycoside hydrolase family 3 protein	GH3	2.992	4.962	30.714*	0.00	0.14	3.32
NRRL3_00268	An09g03300	alpha-xylosidase Axl31A	GH31	1.211	3.397	8.676*	0.00	0.00	0.00
NRRL3_02630	An01g12150	beta-galactosidase Lac35B	GH35	1.589	2.203	33.505*	#N/A	#N/A	#N/A
**NRRL3_02479**	**An01g10350**	**exo-beta-1,4-galactanase**	GH35	3.480	22.294*	24.938*	0.00	1.39	0.00
**NRRL3_06244**	**An02g00140**	**glycoside hydrolase family 43 protein**	GH43	0.812	22.185*	22.908*	#N/A	#N/A	#N/A
NRRL3_04608	An07g04930	glycoside hydrolase family 43 protein	GH43	1.269	2.703	5.432*	#N/A	#N/A	#N/A
NRRL3_06791	An16g06800	glycoside hydrolase family 5 protein	GH5_5	22.647	68.809	665.632*	24.31	128.89	700.71
**NRRL3_10865**	**An08g01710**	**alpha-arabinofuranosidase**	GH51	0.795	11.209*	10.280*	#N/A	#N/A	#N/A
NRRL3_03768	An15g02300	alpha-arabinofuranosidase Abf54B	GH54	0.807	3.673	10.070*	0.38	6.53	2.49
NRRL3_01787	An01g01870	xyloglucanase Xeg74C	GH74	0.055	0.174	4.919*	#N/A	#N/A	#N/A
NRRL3_00701	An14g01330	glycoside hydrolase family 79 protein	GH79	0.070	0.460	8.260*	#N/A	#N/A	#N/A
NRRL3_05305	An02g11890	beta-glucuronidase Gus79A	GH79	0.209	0.447	2.675*	0.00	0.00	0.38
**NRRL3_07382**	**An16g00540**	**alpha-L-fucosidase-like protein**	GH95	0.036	0.667*	2.139*	0.00	0.00	0.32
NRRL3_07089	An16g02760	alpha-L-fucosidase	GH95	1.619	2.893	11.179*	0.00	0.00	0.61

In total, 124 genes were significantly upregulated (FC ≥ 4, FDR ≤ 0.01) in the *OEgaaR* strain compared to the reference strain (Online Resource 4). The promoter regions of 110 upregulated genes for which the *A. niger* CBS 513.88 gene ID was available were screened for the presence of transcription factor binding sites using TFBSF (Meyer et al. 2009), and it was found that 69 genes contain the galacturonic acid-responsive element GARE (CCNCCAA) (Martens-Uzunova and Schaap 2008) required for GA-responsive gene induction (Niu et al. 2015) in their 1 kb upstream sequences. A gene ontology enrichment analysis via FetGOat (Nitsche et al. 2011) indicated that the genes upregulated in the*OEgaaR* strain were highly enriched with genes involved in carbohydrate (xyloglucan, pectin, lactose) metabolism. Out of 53 genes belonging to the GaaR/GaaX panregulon (Niu et al. 2017), 34 were upregulated in the *OEgaaR* strain, including *gaaX* with a fold change of 24.1 (Online Resource 4 and Table 1). Apart from the aforementioned genes, several genes with unknown relation to GA utilization were also upregulated in the *OEgaaR* strain. These include genes encoding hypothetical/uncharacterized proteins, proteins involved in diverse processes such as dehydrogenases and non-ribosomal peptide synthetases, MFS-type transporters and a Zn_2_Cys_6_ type transcription factor (NRRL3_11827) (Online Resource 4).

### Exoproteome analysis of the *OEgaaR* strain

To support the observed transcriptional upregulation of CAZymes in the *OEgaaR* strain, we analyzed the exoproteome of the *OEgaaR* strain and compared it to the exoproteome of the reference and *ΔgaaX* strains grown in bioreactors on fructose (Online Resource 5). Mass-spectrometric analysis revealed 18 pectinases in the exoproteome of the *OEgaaR* strain. Seventeen of them were secreted at higher levels compared to the reference strain and the *ΔgaaX* strain. The protein level of the putative pectin methylesterase NRRL3_05252 was higher in *ΔgaaX* than in the *OEgaaR* strain similar to observed higher mRNA level of this gene in *ΔgaaX* (Table 1). Fifteen of these detected pectinases were also transcriptionally upregulated in the *OEgaaR* strain. In addition, eight genes encoding CAZymes that were expressed at higher levels in the *OEgaaR*strain compared to the reference strain and *ΔgaaX* were found to accumulate at higher levels in the culture media of the *OEgaaR* strain (Table 2). With regard to the degradation of pectin, there is a good correspondence between the upregulated expression of genes and the increased extracellular accumulation of their encoding pectinolytic enzymes in the *OEgaaR* strain, for example the pectinases Pel1A, Pga28E, and Pga28C (Table 1). The distinct SDS-PAGE profile of the *OEgaaR* strain compared to the reference strain and *ΔgaaX* might represent the differences in abundance of the aforementioned extracellular proteins, such as NRRL3_06791 and Pel1A with predicted molecular weights of unglycosylated proteins of 54.8 and 39.7 kDa, respectively (Fig. 1b).

### Nuclear concentration of GaaR is increased in the *OEgaaR* strain

Strains expressing an *eGFP*-tagged *gaaR*, directed by either the endogenous *gaaR* promoter (*eGFP-gaaR*) or the strong constitutive *A. nidulans gpdA* promoter (*OEeGFP-gaaR*), were constructed to investigate the subcellular localization of GaaR in the reference and *OEgaaR* strains, respectively. Expression of eGFP-tagged *gaaR* in a *∆gaaR* background resulted in partial complementation of growth on GA and full complementation of growth on PGA and AP, in both *eGFP-gaaR* (EA19.2) and *OEeGFP-gaaR* (EA20.10) strains (Fig. ESM_3.2 a). The polygalacturonase activity in the culture supernatant of the *OEeGFP-gaaR* strain grown in fructose was higher compared to the *eGFP-gaaR* strain, resembling the polygalacturonase production capacities of the *OEgaaR* and reference strains, respectively (Fig. ESM_3.3 c). These results indicate that the eGFP-tagged GaaR is able to activate the transcription of the GA-responsive genes required for growth on GA-containing carbon sources, and that overexpression of the *eGFP*-tagged *gaaR* results in an increased accumulation of pectinases as in the overexpression of the untagged *gaaR*.

The subcellular localization of GaaR and GaaX was analyzed qualitatively using confocal laser scanning microscopy. As a nuclear marker suitable for co-localization experiments, the SYTO59 dye was used. The nuclear localization of the SYTO59 dye was confirmed in an *A. niger* strain harboring the eGFP-tagged H2B protein (MA26.1) (Fig. ESM_3.4 a). The *eGFP-gaaR*, *OEeGFP-gaaR*, and *gaaX-eGFP* (JN126.2) strains were grown in GA and nuclei were stained with SYTO59 (Fig. ESM_3.4 b). Both eGFP-GaaR and GaaX-eGFP were found to be present in the cytoplasm and nucleus, although we cannot exclude the possibility that eGFP was cleaved off from the fusion proteins and resulted in cytoplasmic or nuclear fluorescence signal. The co-localization experiment showed that eGFP-GaaR was mainly localized in the nucleus in both *eGFP-gaaR* and *OEeGFP-gaaR* strains. In the *gaaX-eGFP* strain grown in GA, GaaX-eGFP was present in both the cytoplasm and the nuclei at roughly the same level.

**Fig. 2:**
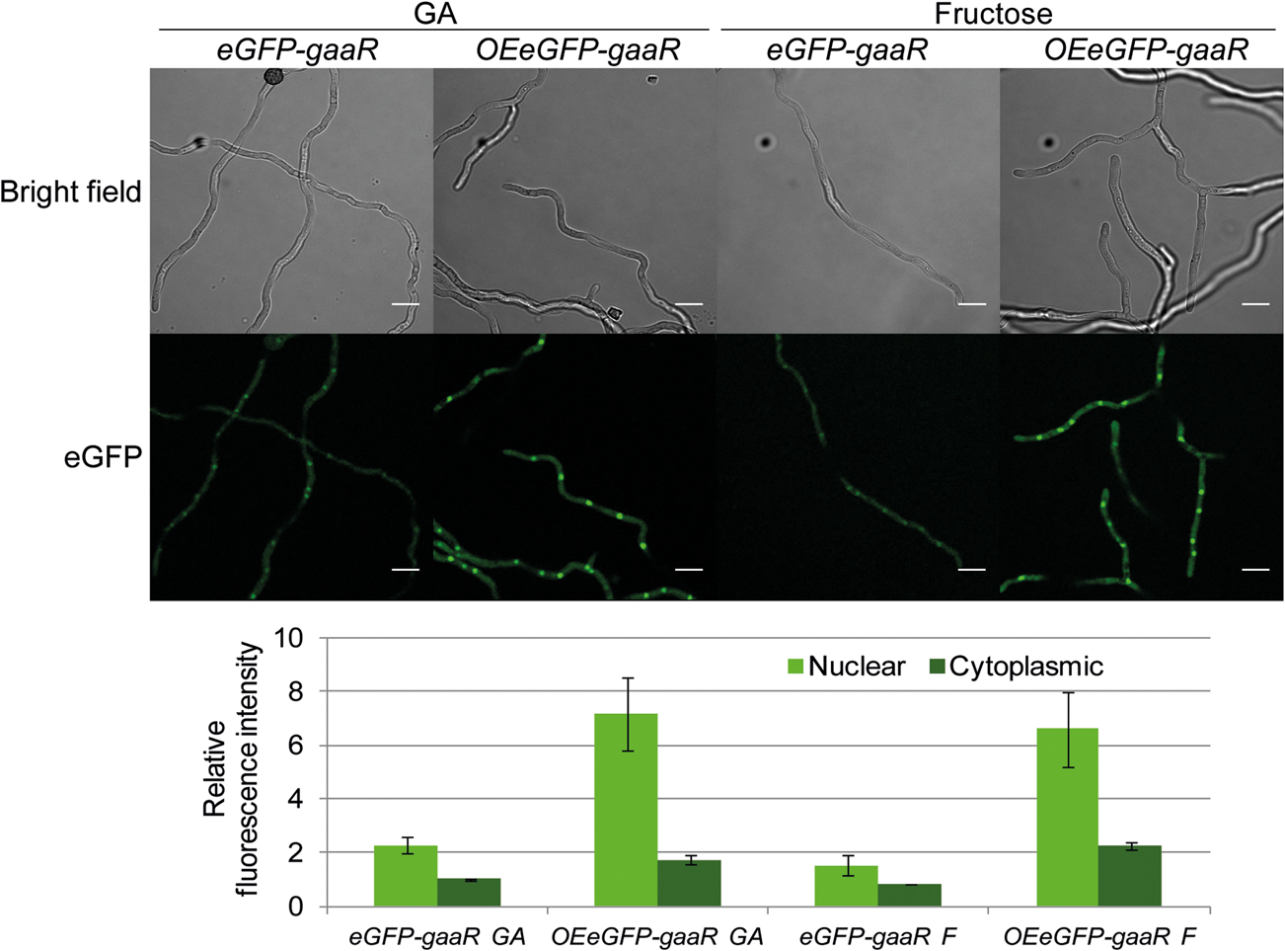
Nuclear and cytoplasmic fluorescence intensity of the eGFP-tagged GaaR protein. The *eGFP-gaaR* (EA19.2) and *OEeGFP-gaaR* (EA20.10) strains were grown in MM containing 50 mM GA and 1 mM fructose, or 50 mM fructose (F) for 17.5 h. Example micrographs representing each condition are shown. Bars represent averages of two biological replicates and standard deviation is shown. Data is represented relative to the cytoplasmic fluorescence intensity in the *eGFP-gaaR* strain on GA. Scale bar 10 μm

We next quantified the cytoplasmic and nuclear eGFP-GaaR intensity in the *eGFP-gaaR* and *OEeGFP-gaaR* strains grown in GA or fructose. As shown in Fig. 2, nuclear eGFP-GaaR fluorescence was higher than the cytoplasmic intensity regardless of the presence of an inducing carbon source or the promoter used to overexpress *eGFP*-tagged *gaaR*. The GFP signals in the *eGFP-gaaR* strain were low after growth in GA or fructose, confirming previous findings that *gaaR* is expressed at low levels on both GA and fructose (Alazi et al. 2016 and Table 1). Overexpression of *eGFP-gaaR* resulted in a much higher nuclear eGFP-GaaR concentration in the *OEeGFP-gaaR* strain than in the *eGFP-gaaR* strain, while only a slight increase in the cytoplasmic concentration was observed. This indicates that the excess eGFP-GaaR produced in the *OEeGFP-gaaR* strain localizes mainly in the nucleus. This result is in line with the observation that in a *Botrytis cinerea* strain overexpressing *BcgaaR-eGFP*, BcGaaR-eGFP mainly localizes in the nucleus under inducing or non-inducing conditions (Zhang et al. 2016).

### Deletion of *creA* in the *OEgaaR* strain results in elevated production of pectinases

**Fig. 3:**
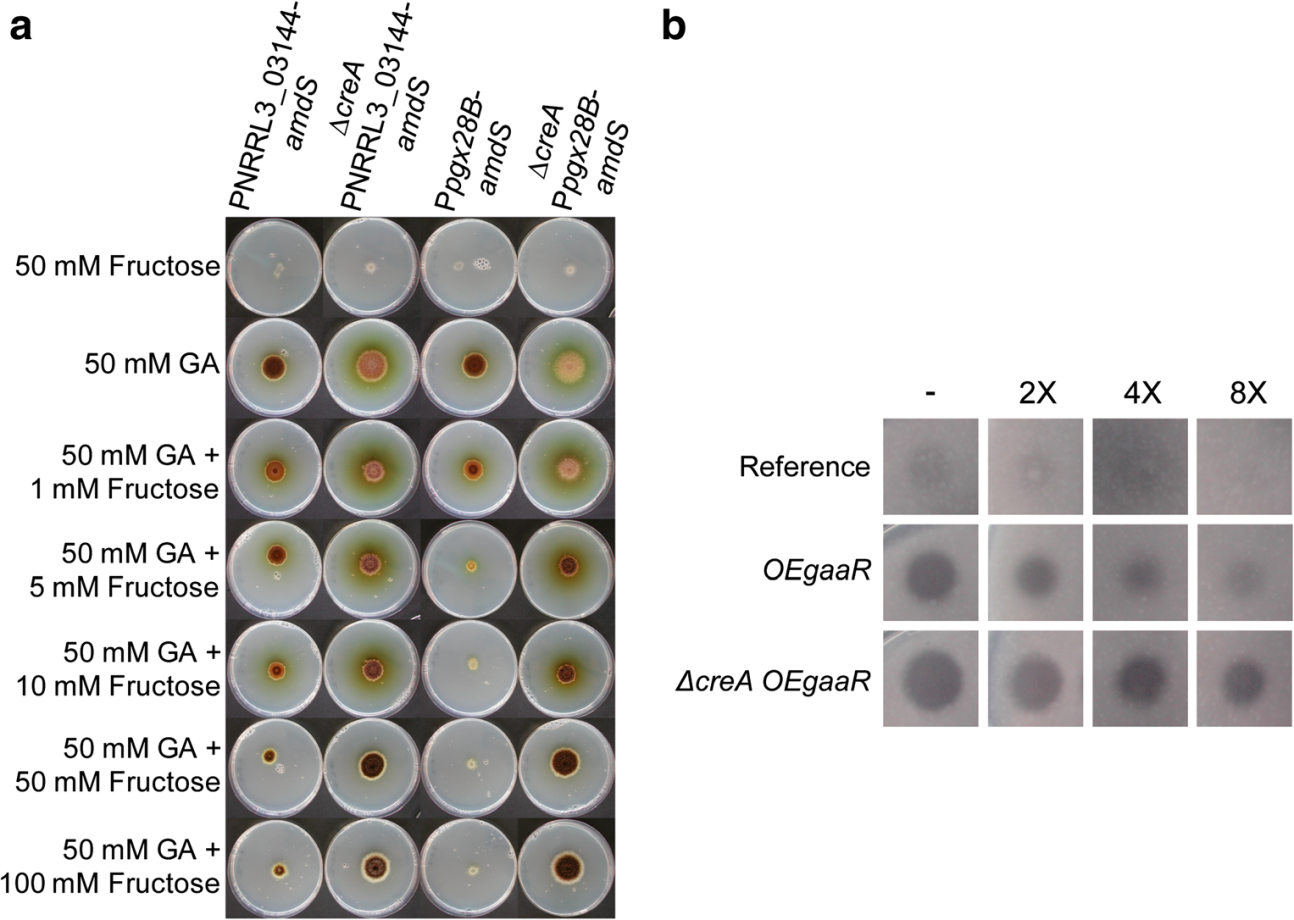
Analysis of CreA-mediated carbon catabolite repression on pectinase genes. **a** Growth phenotype of the PNRRL3_03144-*amdS* (JC1.5), *∆creA* PNRRL3_03144-*amdS* (JN29.2), P*pgx28B*-*amdS* (JC3.6) and *∆creA* P*pgx28B*-*amdS* (JN31.3) strains on solid MM containing 50 mM fructose, 50 mM GA, or 50 mM GA with increasing amounts of fructose after 7 days at 30 °C. All plates contain 10 mM acetamide as the sole nitrogen source. **b** PGA plate assay. The reference (MA234.1), *OEgaaR* (EA21.6), and *∆creAOEgaaR* (TK2.1) strains were grown in MM containing 50 mM fructose for 36 h, and serial dilutions of culture supernatants were spotted on PGA plates. Dilution factors are indicated

Carbon catabolite repression in the presence of glucose on several GA-responsive genes encoding exo-polygalacturonases, e.g., NRRL3_03144 (*pgaX*) and *pgx28B* (*pgxB*), was previously shown to be CreA-mediated (Niu et al. 2015). The *OEgaaR* strains produced more polygalacturonases during growth in fructose compared to glucose, indicating that fructose exerts less repression than glucose on pectinase gene expression (Fig. ESM_3.2 a). To investigate to which extent the presence of fructose affects CreA-mediated carbon catabolite repression on pectinase gene expression, we used promoter-reporter strains PNRRL3_03144-*amdS* and P*pgx28B*-*amdS*, which are able to grow on acetamide as the sole nitrogen source only when the *amdS* gene encoding the acetamidase enzyme is expressed via the GA-responsive promoters of the pectinase genes NRRL3_03144 and *pgx28B*, respectively (Niu et al. 2015). Growth of the promoter-reporter strains on plates containing acetamide and GA decreased as the fructose concentration in the growth media increased (Fig. 3a), indicating that fructose also represses the expression of genes encoding those pectinases. In addition, we directly compared the repression power of glucose, fructose, sorbitol, and acetate on NRRL3_03144 expression in a single experiment (Fig.ESM_3.5). Radial growth assay confirmed that the expression NRRL3_03144 is repressed strongly by glucose and mildly by fructose. Sorbitol and acetate exerted negligible repression on NRRL3_03144 (Fig. ESM_3.5). Deletion of *creA* restored the growth of the promoter-reporter strains on fructose, showing that fructose-imposed carbon catabolite repression on pectinase gene expression is also mediated by CreA (Fig. 3a).

All 124 genes that were upregulated in the *OEgaaR* strain in fructose and the promoter regions of which could be screened for the presence of transcription factor binding sites, contain at least one CreA binding motif (SYGGRG) (Cubero and Scazzocchio 1994) in their 1 kb upstream sequences (Online Resource 4), suggesting that carbon repression has a major effect on the expression on these GaaR target genes. Because the presence of fructose has a repressing effect on the expression of pectinase genes such as exo-polygalacturonases, NRRL3_03144 and *pgx28B*, we hypothesized that deletion of *creA* in the *OEgaaR* background would result in an elevated expression of pectinase genes on fructose. Therefore, the *∆creAOEgaaR* strain (TK2.1) was created in the EA21.6 background to allow a direct comparison. Growth analysis on plates showed a reduced growth of the *∆creAOEgaaR* strain on glucose, fructose, and AP, which was also observed in a control *∆creA* strain (MA342.2) indicating that the reduced growth is caused by the *creA* deletion and not by *gaaR* overexpression (ESM_3.2 a).To assess the effect of *creA* deletion on pectinase production in combination with *gaaR* overexpression, the reference strain (MA234.1), *OEgaaR* (EA21.6) and *∆creAOEgaaR* (TK2.1) strains were grown in fructose and the polygalacturonase activity in the culture supernatants was analyzed via a PGA plate assay. The culture supernatant of the *∆creAOEgaaR* strain displayed the highest polygalacturonase activity, thereby providing additional evidence that fructose exerts repression on pectinase gene expression through CreA (Fig. 3b).

## Discussion

The Zn_2_Cys_6_ transcriptional activator GaaR (Alazi et al. 2016) and the repressor protein GaaX (Niu et al. 2017) are the two important players in the transcriptional regulation of the GA-responsive genes in *A. niger*. Both GaaR and GaaX are highly conserved in filamentous fungi of the phylum ascomycetes. Therefore, the GaaR/GaaX module is expected to be the main regulatory mechanism in controlling GA-induced gene expression in filamentous fungi of ascomycetes. The combination of an activator (GaaR) and repressor (GaaX) protein to control gene expression represents a conserved mechanism which shows striking similarities with the regulation of genes involved in quinic acid utilization in *A. nidulans* (Lamb et al. 1996). In both the regulation of GA-responsive genes as well as in quinic acid-responsive genes, loss of function of the respective repressor proteins results in constitutive and inducer-independent expression of target genes. Importantly, the induced expression still requires the corresponding transcriptional activator (Grant et al. 1988; Niu et al. 2017). These observations suggest a model in which the transcriptional activator is kept inactive by its corresponding repressor protein under non-inducing conditions. Upon inducing conditions, an inducer molecule is expected to bind to the repressor thereby causing its dissociation from the transcriptional activator. Non-repressor bound activator is expected to be active as a transcription factor to induce the expression of target genes (Lamb et al. 1996; Niu et al. 2017).

In this study, we constructed several *A. niger* strains that overexpress *gaaR* via the *A. nidulans gpdA* promoter. The *OEgaaR* strains, carrying different copy numbers of the ectopically integrated *gaaR* overexpression construct, showed partial and different levels of complementation of growth on GA-containing carbon sources, whereas their growth on glucose or fructose was similar to the reference strain. While in the wild type high levels of pectinases are produced only under inducing conditions, the *OEgaaR* strains secreted high levels of polygalacturonases under both inducing and non-inducing conditions. These results imply that the *OEgaaR* strains possess a functional GaaR that is able to activate the expression of genes required for growth on GA and genes encoding polygalacturonases. Among all *OEgaaR* strains, EA21.6 and TK1.1 displayed the most impaired growth on GA, PGA, and AP and produced the highest levels of polygalacturonases in fructose. This might indicate a possible cofactor imbalance due to increased amounts of NAD(P)H-dependent GaaA and NADPH-dependent GaaD enzymes, or accumulation of a toxic GA catabolic pathway intermediate, when *OEgaaR* strains grow on GA-rich carbon sources.

The GA-responsive genes are transcriptionally induced by GaaR under inducing conditions (Alazi et al. 2016; Martens-Uzunova and Schaap 2008) and the transcriptional activity of GaaR is suggested to be controlled by GaaX, possibly via a protein-protein interaction, under non-inducing conditions (Niu et al. 2017). We showed that eGFP-GaaR is localized mainly in the nucleus under both inducing and non-inducing conditions, indicating that the transcriptional activity of GaaR is not regulated through nuclear translocation upon induction and the mechanism which keeps GaaR inactive under non-inducing conditions is likely to occur in the nucleus. Prediction of nuclear localization signals using the prediction tool NucPred (Brameier et al. 2007) indicated that GaaR likely localizes in the nucleus (score of 0.90) whereas GaaX (score of 0.27) is expected to spend less time in the nucleus. Nevertheless, GaaX-eGFP was found to be present in both cytoplasm and nucleus under inducing conditions, showing that it can enter the nucleus (Fig. ESM_3.4 b). These results imply that GaaX might inhibit the transcriptional activity of GaaR in the nucleus under non-inducing conditions.

Ectopic integration of *gaaR* in a *ΔgaaR* strain was previously shown to result in a full complementation of growth on GA (Alazi et al. 2016), whereas the *eGFP-gaaR* strain EA19.2 that was derived from a *ΔgaaR* strain and expresses N-terminally eGFP-tagged *gaaR* displayed a slightly reduced growth on GA compared to the reference strain. N-terminal eGFP-tagging might result in a minor decrease in GaaR transcription factor activity and therefore partial restoration of growth. As GaaR is expected to interact with GaaX, it was also assessed whether the N-terminal tagging of GaaR influenced its interaction with GaaX. The expression of *eGFP-gaaR* via the endogenous *gaaR* promoter did not result in a constitutive expression of the genes encoding polygalacturonases (Fig. ESM_3.3 c), indicating that eGFP-GaaR activity is properly controlled by GaaX under non-inducing conditions.

Overexpression of eGFP-GaaR driven by the *A. nidulans gpdA* promoter leads to a much higher nuclear eGFP-GaaR concentration under both inducing and non-inducing conditions compared to expression driven by the endogenous *gaaR* promoter. The increase in nuclear GaaR concentration was accompanied by transcriptional upregulation of GA-responsive genes and the increased accumulation of pectinases in the extracellular medium. The transcriptional activation of GA-responsive genes in the *OEgaaR* strain under non-inducing conditions can be explained by the possibility that the excess of GaaR titrates out the concentration of GaaX and escapes GaaX inhibition, even though *gaaX* is induced upon GaaR overexpression.

Genome-wide gene expression analysis in the reference strain grown in GA has been previously performed (Alazi et al. 2016). Direct comparison of the gene expression values between the study of Alazi et al. (2016), and this study needs careful interpretation due to different experimental setups (growth in shake flasks vs bioreactors) and representation of transcript levels (FPKM vs TPM). Notwithstanding, it can be observed that the expression level of the genes encoding pectinases are generally comparable between the reference and *OEgaaR* strains grown under inducing and non-inducing conditions, respectively. However, drastically higher expression of NRRL3_05252, NRRL3_03144, *pgx28B*, and *gan53A* in the reference strain and *pga28C*, *pga28E*, and *pel1A* in the *OEgaaR* strain was also observed.

Elimination of the repressing activity of GaaX by deleting *gaaX* was previously shown to be another way to activate the expression of GA-responsive genes under non-inducing conditions (Niu et al. 2017). The concentration of the nuclear GaaR in *ΔgaaX* is expected to be similar to wild type and much less compared to the *OEgaaR* strain. Only nine out of 48 genes encoding pectinases were upregulated in the *ΔgaaX* strain in fructose compared to the reference strain, and the transcript and extracellular protein levels of these pectinases were generally lower compared to the *OEgaaR* strain. This indicates that the nuclear concentration of active GaaR is indeed an important factor for transcriptional activation of GA-responsive genes. On the other hand, the genes encoding the (putative) GA transporters and catabolic pathway enzymes were expressed at higher levels in *ΔgaaX* compared to the *OEgaaR* strain, indicating that factors other than GaaR concentration might play a role in the regulation of these genes.

RNA-seq analysis showed that besides the genes encoding pectinases, 20 genes predicted to encode CAZymes involved in the degradation of multiple substrates or specifically of cellulose, starch, or xyloglucan were upregulated in the *OEgaaR* strain in fructose. This indicates that these enzymes might be involved or assist in enabling the degradation of pectin. Five of these CAZymes were shown to be upregulated in *ΔgaaX*, and therefore designated as part of the GaaR/GaaX panregulon (Niu et al. 2017). In addition, many of the 20 additional CAZymes were reported to be potentially regulated by transcription factors AraR and/or XlnR (Gruben et al. personal communication). However, the expression of the genes encoding AraR or XlnR were not significantly changed in *OEgaaR*, discounting the possibility that overexpression of GaaR caused transcriptional upregulation of the genes encoding CAZymes via their specific transcription factors.

Fructose was found to exert CreA-mediated repression of gene expression in case of the genes NRRL3_03144 (*pgaX*) and *pgx28B* (*pgxB*) encoding exo-polygalacturonases, which were previously shown to be strongly repressed in the presence of glucose (Niu et al. 2015). The repression power of fructose was lower than that of glucose (Fig. ESM_3.5). As shown by Niu et al. (2017), deletion of *creA* is not sufficient for an increased production of polygalacturonases under non-inducing conditions, showing that GA-responsive gene expression requires the presence of active GaaR relieved from GaaX inhibition. A similar phenomenon was previously observed in *T. reesei*, where high expression of the genes encoding cellulases in a Cre1-disrupted strain required the presence of the transcriptional activator Xyr1 under non-inducing conditions (Wang et al. 2013). The strain that lacks *creA* and overexpresses GaaR (TK2.1) secreted higher levels of polygalacturonases compared to the reference and *OEgaaR* strains, indicating that CreA substantially represses the expression of GA-responsive genes in the presence of fructose even when GaaR is abundant.

To conclude, genetic evidence suggests that the activity of the GaaR transcription factor is negated by the action of the GaaX repressor protein. Either deletion of GaaX or overexpression of GaaR results in a constitutive expression of GaaR/GaaX target genes. The simplest interpretation of these observations is that GaaX mediates its repressing activity by a direct interaction with GaaR. Loss of function of GaaX or overexpression of GaaR will affect the stoichiometry of GaaR-GaaX and lead to high levels of “repressor free” GaaR which is expected to act as an active transcription factor to induce expression of GA-responsive genes. We have shown that overexpression of GaaR leads to an increased level of pectinase production under non-inducing conditions, and that deletion of *creA* further increases the pectinase production capacity of *A. niger*. The *∆creAOEgaaR* strain represents an interesting strain for applications in industry with its high pectinase production capacity in the absence of an inducing carbon source and in the presence of a repressing carbon source.

## Electronic supplementary material

ESM 1 (PDF 977 kb)

ESM 2 (XLSX 1425 kb)
