# Supplementary material for: Inducer-independent production of pectinases in Aspergillus niger by overexpression of the D-galacturonic acid-responsive transcription factor gaaR
Source: Appl Microbiol Biotechnol. 2018 Jan 24;102(6):2723–36. doi: 10.1007/s00253-018-8753-7 (PMC5847190; doi:10.1007/s00253-018-8753-7)
Supplement: Supplementary file 1 — (PDF 977 kb) [file 253_2018_8753_MOESM1_ESM.pdf]

Applied Microbiology and Biotechnology

Inducer-independent production of pectinases in *Aspergillus niger* by overexpression of the D-galacturonic acid responsive transcription factor *gaaR*

Ebru Alazi[1], Tim Knetsch[1], Marcos Di Falco[2], Ian D. Reid[2], Mark Arentshorst[1], Jaap Visser[1], Adrian Tsang[2] and Arthur F.J. Ram[1]

[1] Molecular Microbiology and Biotechnology, Institute of Biology Leiden, Leiden University, Sylviusweg 72, 2333 BE Leiden, The Netherlands

[2] Centre for Structural and Functional Genomics, Concordia University, 7141 Sherbrooke Street West, Montreal, Quebec H4B1R6, Canada

E-mail: a.f.j.ram@biology.leidenuniv.nl Tel: +31 71 527 4914 Fax: +31 71 527 4900

ESM_1 Strains used in this study

Strain	Accession Number	Genotype	Description	Reference
N402	ATCC 64974	*cspA1*	Derivative of NRRL3 (N400)	Bos et al. 1988
MA234.1	CBS 141255	*cspA1, kusA::DR-amdS-DR*	Restored *pyrG* in MA169.4	Alazi et al. 2016
XY1.1	CBS 143276	*cspA1, creA::pyrG*	*ΔcreA* in AB4.1	Yuan et al. 2006
MA342.2	CBS 143258	*cspA1, kusA::DR-amdS-DR, creA::hygB*	*ΔcreA* in MA234.1	This study
JN35.1	CBS 141256	*cspA1, kusA::DR-amdS-DR, gaaR::hygB*	*ΔgaaR* in MA234.1	Alazi et al. 2016
JN36.1	CBS 143259	*cspA1, gaaR::hygB*	*amdS* loopout in JN35.1	Alazi et al. 2016
EA21.3	CBS 143260	*cspA1, gaaR::hygB, amdS*$^{+}$*, ectopically integrated* P*gpdA-gaaR*	*gaaR* overexpression in JN36.1	This study
EA21.5	CBS 143261	*cspA1, gaaR::hygB, amdS*$^{+}$*, ectopically integrated* P*gpdA-gaaR*	*gaaR* overexpression in JN36.1	This study
EA21.6	CBS 143262	*cspA1, gaaR::hygB, amdS*$^{+}$*, ectopically integrated* P*gpdA-gaaR*	*gaaR* overexpression in JN36.1	This study
EA21.8	CBS 143263	*cspA1, gaaR::hygB, amdS*$^{+}$*, ectopically integrated* P*gpdA-gaaR*	*gaaR* overexpression in JN36.1	This study
TK1.1	CBS 143264	*cspA1, gaaR::hygB, amdS*$^{+}$*, ectopically integrated* P*gpdA-gaaR*	*gaaR* overexpression in JN36.1	This study
EA23.6	CBS 143265	*cspA1, gaaR::hygB, amdS*$^{+}$*, ectopically integrated* P*gpdA-gaaR, pyrE*$^{-}$	Loss of *pyrE* in EA21.6	This study
TK2.1	CBS 143266	*cspA1, gaaR::hygB, amdS*$^{+}$*, ectopically integrated* P*gpdA-gaaR, pyrE*$^{-}$*, ΔcreA::pyrF*	*ΔcreA* in EA23.6	This study
EA19.2	CBS 143267	*cspA1, gaaR::hygB, amdS*$^{+}$*, ectopically integrated* P*gaaR-eGFP-gaaR*	*eGFP-gaaR* in JN36.1	This study
EA20.10	CBS 143268	*cspA1, gaaR::hygB, amdS*$^{+}$*, ectopically integrated* P*gpdA-eGFP-gaaR*	*eGFP-gaaR* overexpression in JN36.1	This study
JN126.2	CBS 143269	*cspA1, Δku70::amdS, gaaX::nicB,* P*gaaX-gaaX-eGFP* integrated to the *pyrG* locus	*gaaX-eGFP* in JN125.1	Niu et al. 2017
MA26.1	CBS 143270	*cspA1, amdS*$^{-}$*,* P*gpdA-H2B-eGFP*	*H2B-eGFP* overexpression in AB4.1	Vinck, 2007
JC1.5	CBS 143271	*cspA1, Δku70,* PNRRL3_03144-*amdS* integrated to the *pyrG* locus	PNRRL3_03144-*amdS* in MA299.2	Niu et al. 2015
JN29.2	CBS 143272	*cspA1, Δku70,* PNRRL3_03144-*amdS* integrated to the *pyrG* locus*, creA::hygB*	*ΔcreA* in JC1.5	Niu et al. 2015
JC3.6	CBS 143273	*cspA1, Δku70,* P*pgx28B-amdS* integrated to the *pyrG* locus	P*pgx28B-amdS* in MA299.2	Niu et al. 2015
JN31.3	CBS 143274	*cspA1, Δku70,* P*pgx28B-amdS* integrated to the *pyrG* locus*, creA::hygB*	*ΔcreA* in JC3.6	Niu et al. 2015
JN123.1	CBS 143275	*cspA1, Δku70,* PNRRL3_03144-*amdS* integrated to the *pyrG* locus*, gaaX::hygB*	*ΔgaaX* in JC1.5	Niu et al. 2017

ESM_2 Primers used in this study.

Primer name	Sequence (5' to 3')	Used for	Remarks
gaaR comp P1 PscI	CATGACATGTCCCGCCCCCAGGT	amplification of *gaaR* for the construction of pEA4	*Psc*I site underlined
gaaR comp P2 BglII	GAAGATCTCAAGGATTCTCCACCTCCA	amplification of *gaaR* for the construction of pEA4	*Bgl*II site underlined
gaaR SBfor	CCTCGACGCCATTCCAGTT	amplification of Southern blot probe	
gaaR SBrev	GGTCATGGACACCGCATTG	amplification of Southern blot probe	
gaaR comp P1	**ATGTCCCGCCCCCAGGT**	amplification of *gaaR* for the construction of pEA2 and pEA3	Fusion PCR overlapping region in bold
gaaR comp P2 BglII	GAAGATCTCAAGGATTCTCCACCTCCA	amplification of *gaaR* for the construction of pEA2 and pEA3	*Bgl*II site underlined
eGFP P1 NcoI	GATGCCATGGTGAGCAAGGGCGAG	amplification of *eGFP* for the construction of pEA2 and pEA3	*Nco*I site underlined
eGFP P2 gaaR phu	**CTGACCTGGGGGCGGGACAT**CTTGTACAGCTCGTCCATG	amplification of *eGFP* for the construction of pEA2 and pEA3	Fusion PCR overlapping region in bold
PgaaR P1 NotI	AAGGAAAAAAGCGGCCGCTGGGATTGAAGATGTCGATGC	amplification of P*gaaR* for the construction of pEA2	*Not*I site underlined
PgaaR P2 NcoI	CATGCCATGGCATTGCCTGTGCATAGG	amplification of P*gaaR* for the construction of pEA2	*Nco*I site underlined
creA sm p1f	AAGCAGCCGATCTGGTTCAA	amplification of *creA* 5' flank to create TK2.1	
creA sm p2r	**CAATTCCAGCAGCGGCTT**GTGAAGCTTGTCCCAAGAC	amplification of *creA* 5' flank to create TK2.1	Fusion PCR overlapping region in bold
creA sm p3f	**ACACGGCACAATTATCCATCG**TTCGAACATTCTTCAGCCACAC	amplification of *creA* 3' flank to create TK2.1	Fusion PCR overlapping region in bold
creA sm p4r	GGGAATGGTCTGGTCTCCGT	amplification of *creA* 3' flank to create TK2.1	
Anid pyrFP1for	**AAGCCGCTGCTGGAATTG**ACTGATTGCGC ACATTGAC	amplification of *A. nidulans pyrF* fragment 1 to create TK2.1	Fusion PCR overlapping region in bold
Anid pyrFP4rev	CGCCCTCCTTCTCGATGAT	amplification of *A. nidulans pyrF* fragment 1 to create TK2.1	
Anid pyrFP3for	GCCGTGAATCGCCCTACTT	amplification of *A. nidulans pyrF* fragment 2 to create TK2.1	
Anid pyrFP2rev	**CGATGGATAATTGTGCCGTG**ATCAGGCACGGTCAGTCCTC	amplification of *A. nidulans pyrF* fragment 2 to create TK2.1	Fusion PCR overlapping region in bold
An02g03830P1f_KIT	AGTATTCCACTCAGCTCTGGACTAACCTCTTTAATTCTACTCCG TAATCCCTCTCGAC	amplification of *creA* 5' flank to create MA342.2	
An02g03830P2r_KIT	TGATGTGTGTGACGTGATGTGTATATCCTATCCCAAGACCGAC GAGGGTAAAA	amplification of *creA* 5' flank to create MA342.2	
An02g03830P3f_KIT	TGATGATGGATATATGGAAGCTGGAGGATGTTCGAACATTCT TCAGCCACACGTTG	amplification of *creA* 3' flank to create MA342.2	
An02g03830P4r_KIT	TAGCAGGTGAGAACTCACTTGTACTCGACTCCTGCATACTGGT ATATACAGTAAAACCCCATACTATC	amplification of *creA* 3' flank to create MA342.2	
An02g03830P5f_KIT	ACACCCTCTCTTCCCGGTCA	amplification of split marker fragment 5' to create MA342.2	
An02g03830P6r_KIT	AGTCAATCAAGTAAGTCCCC	amplification of split marker fragment 3' to create MA342.2	
hygP3f	TAGGATATACACATCACGTC	amplification of *hygR* selection marker	
hygP4r	CATCCTCCAGCTTCCATATATC	amplification of *hygR* selection marker	

hygP8f	AAAGTTCGACAGCGTCTCC	amplification of split marker fragment 3’ to create MA342.2
hygP9r	GGCGTCGGTTTCCACTATC	amplification of split marker fragment 5’ to create MA342.2

ESM_3 Supplementary figures

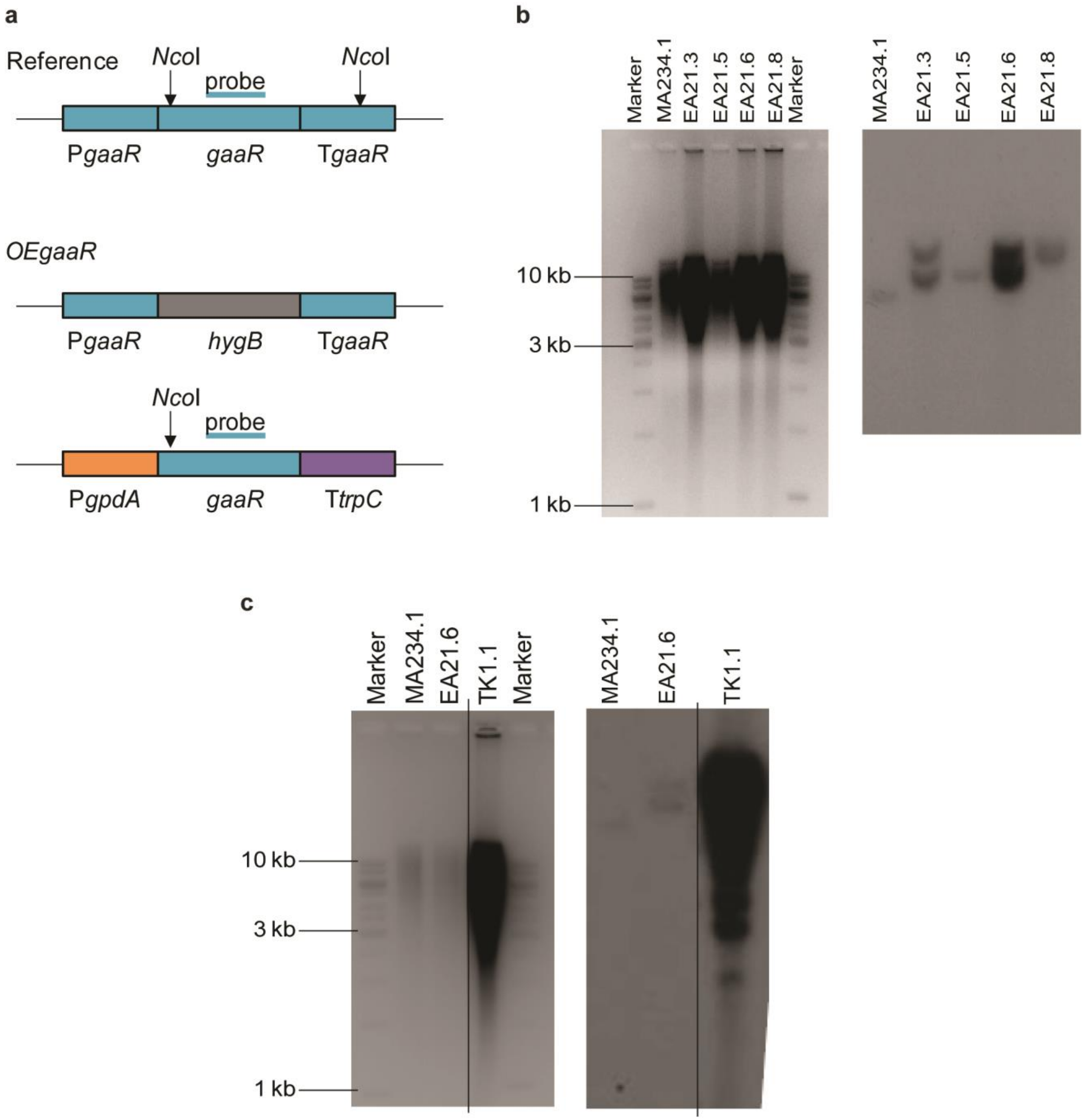

Fig ESM_3.1 Verification of the ectopic integration of the P*gpdA*-*gaaR*-T*trpC* construct in EA21.3, EA21.5, EA21.6 and EA21.8 via Southern blot analysis of genomic DNA. **a** Schematic representation of the target gene locus in the reference (MA234.1) and *OEgaaR* (EA21.3, EA21.5, EA21.6, EA21.8 and TK1.1) strains. The probe binds to *gaaR* downstream of the *Nco*I restriction site. **b** Agarose gel stained with ethidium bromide and Southern blot after hybridization for MA234.1, EA21.3, EA21.5, EA21.6 and EA21.8 strains. c Agarose gel stained with ethidium bromide and Southern blot after hybridization for MA234.1, EA21.6 and TK1.1 strains. The line between genomic DNA samples indicate that left and right parts of the same blot were combined, removing unnecessary lanes. A 4698 bp band is visible in the case of the

reference strain and bands with different sizes are visible in the case of the *OEgaaR* strains depending on the integration site of the *gaaR* overexpression construct in their genomes

a

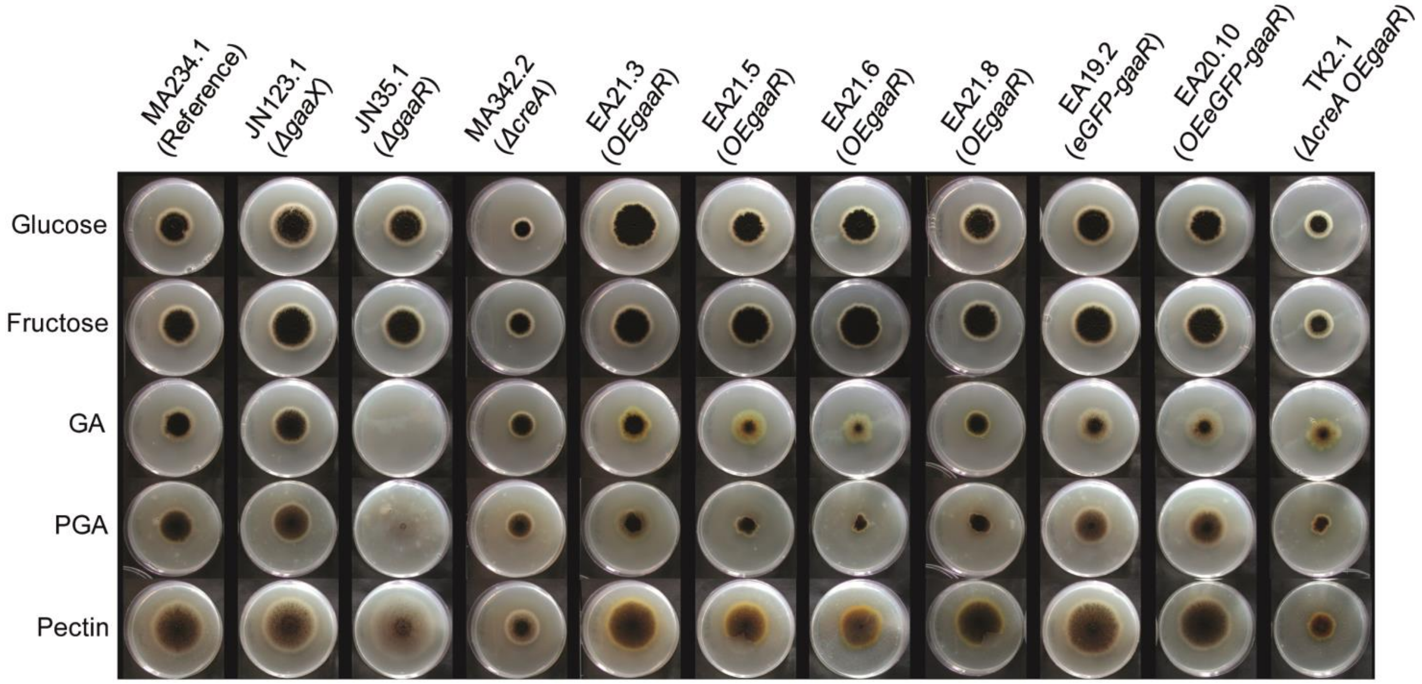

b

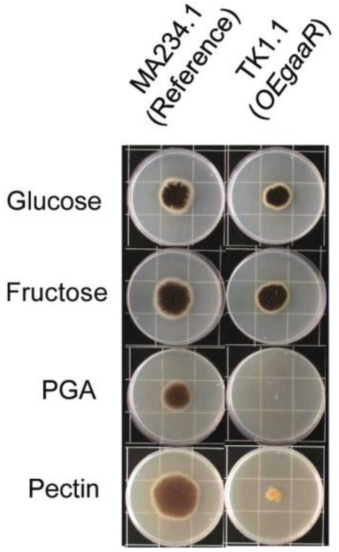

Fig ESM_3.2 Radial growth assay on solid MM containing 50 mM monomeric or 1% polymeric carbon sources after 7 days at 30 °C

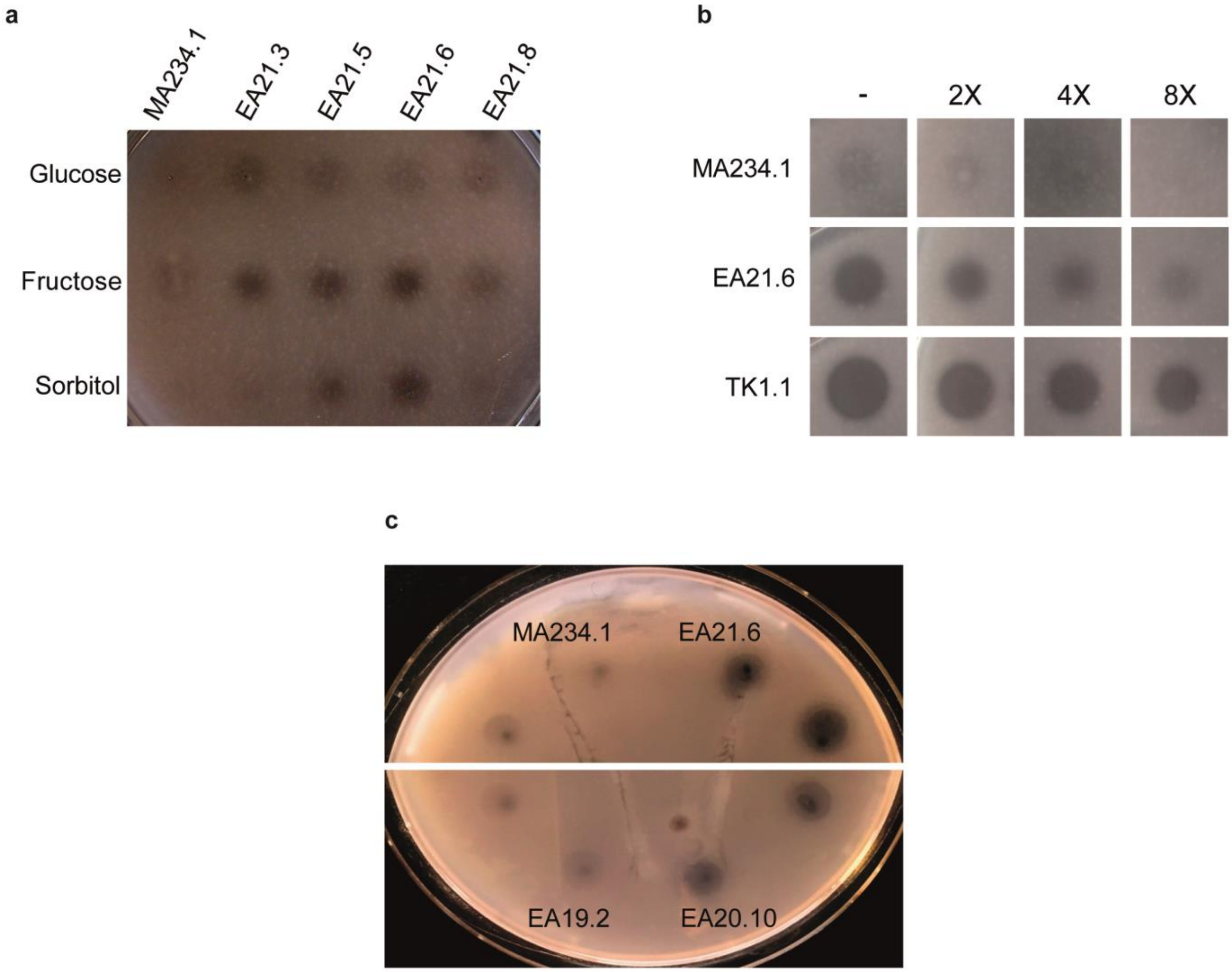

Fig ESM_3.3 PGA plate assay. The reference (MA234.1), *OEgaaR* (EA21.3, EA21.5, EA21.6, EA21.8 and TK1.1), *eGFP-gaaR* (EA19.2) and *OEeGFP-gaaR* (EA20.10) strains were grown in liquid medium for 36 h and supernatant from each culture was spotted on PGA plates. **a** Strains were grown in MM containing 50 mM glucose, fructose or sorbitol. **b** Strains were grown in MM containing 50 mM fructose, and serial dilutions of culture supernatants were spotted. Dilution factors are indicated. **c** Strains were grown in MM containing 50 mM fructose. Enzymatic activity in the supernatants from duplicate cultures are shown

a

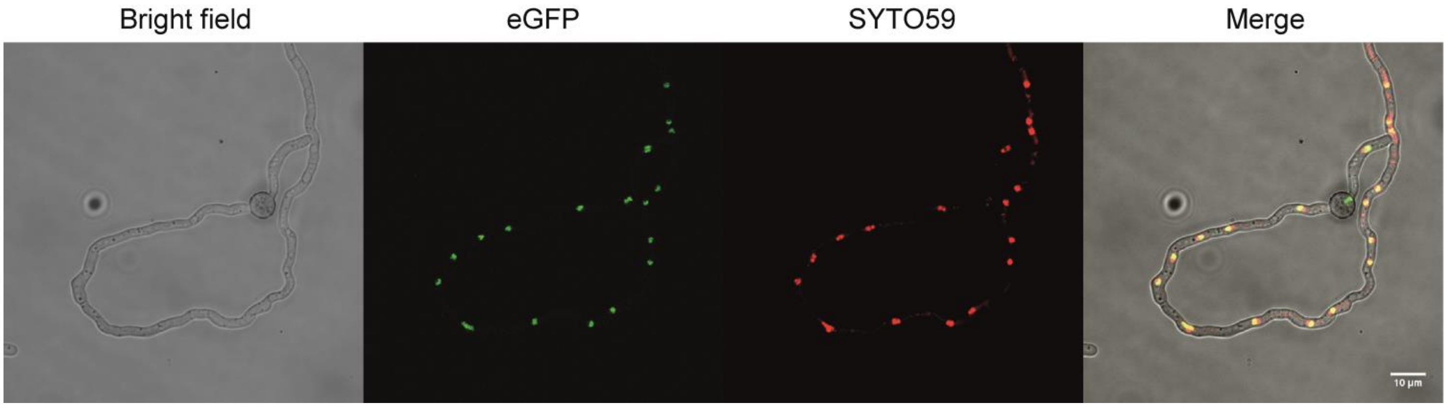

b

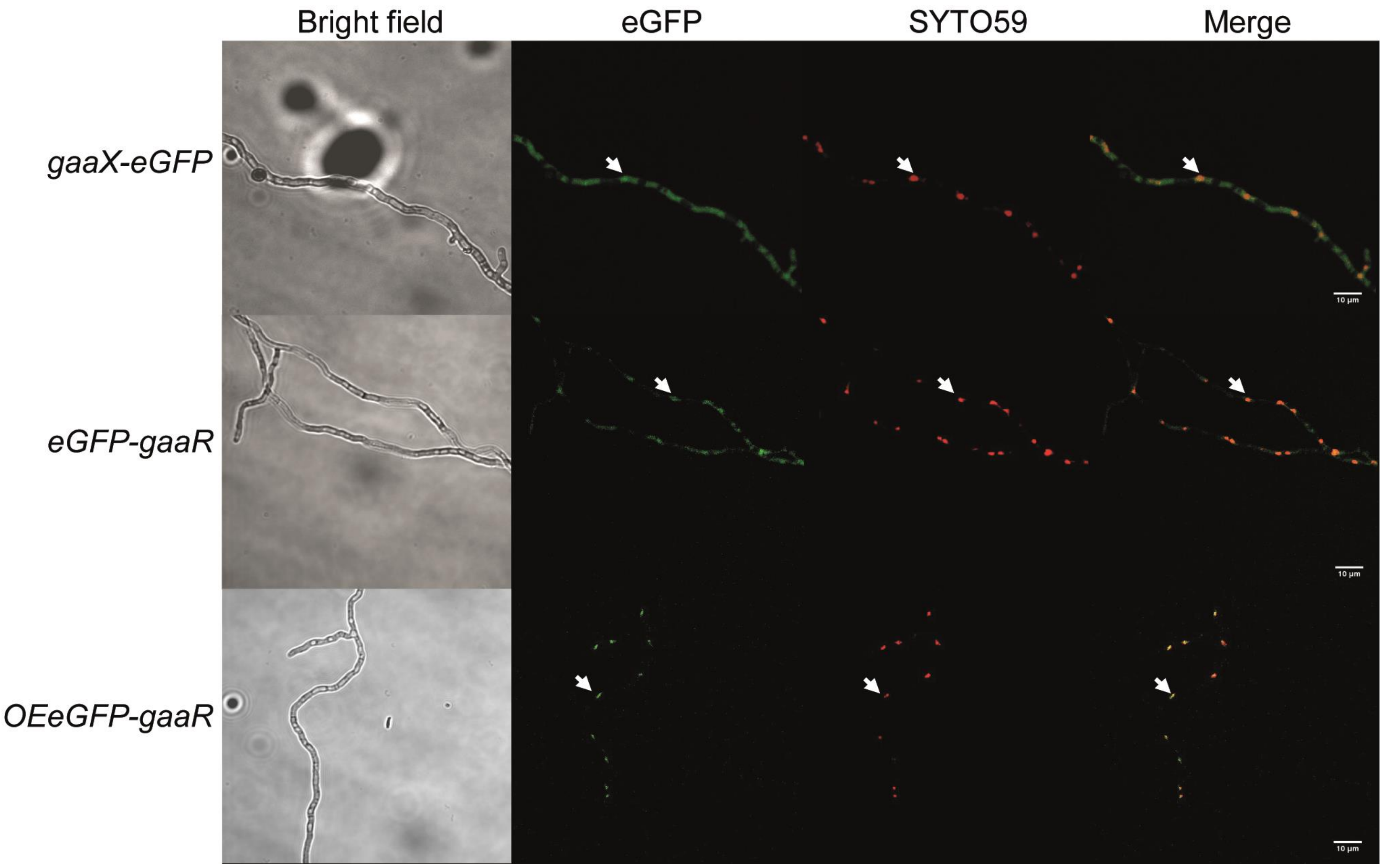

Fig ESM_3.4 a Co-localization of the nuclear specific SYTO59 dye with the eGFP-tagged H2B protein. The MA26.1 strain was grown in MM containing 50 mM fructose for 16 h, and transferred to and grown in MM containing 50 mM GA for 1.5 h. **b** Co-localization of the nuclear specific SYTO59 dye with the eGFP-tagged GaaX and GaaR proteins. The *gaaX-eGFP* (JN126.2), *eGFP-gaaR* (EA19.2) and *OEeGFP-gaaR* (EA20.10) strains were grown in MM containing 10 mM GA for approximately 22 h. Positions of example nuclei were indicated with arrows. Scale bar: 10 µm

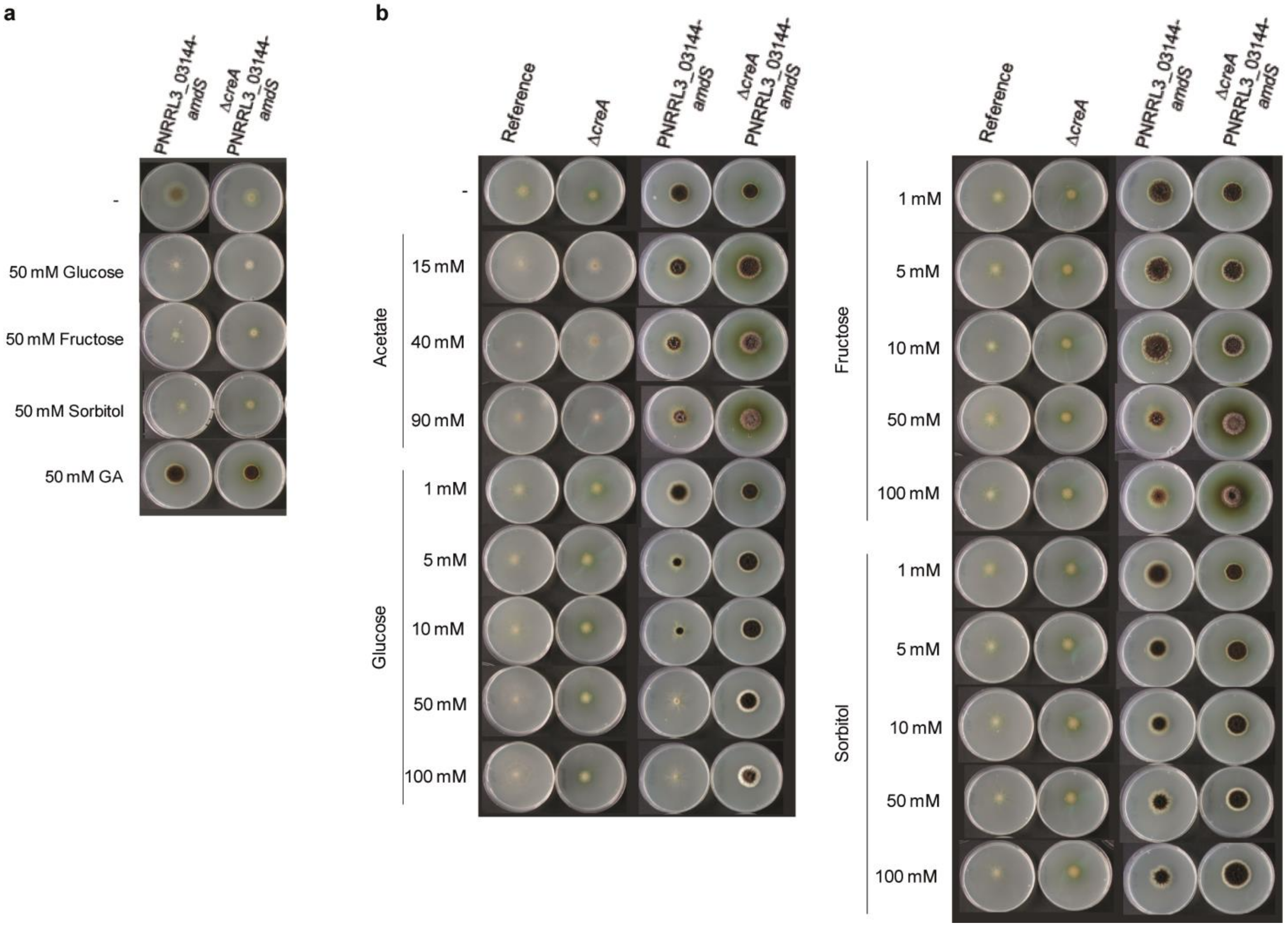

Fig ESM_3.5 Analysis of CreA-mediated carbon catabolite repression on exopolygalacturonase encoding gene NRRL3_03144. The reference (N402), *ΔcreA* (XY1.1), PNRRL3_03144-*amdS* (JC1.5) and *ΔcreA* PNRRL3_03144-*amdS* (JN29.2) strains were grown at 30 °C for 7 days on solid MM containing 50 mM glucose, fructose, sorbitol or GA as carbon source (**a**), or 50 mM GA with increasing amounts of acetate, glucose, fructose or sorbitol (**b**). All plates contain 10 mM acetamide as the sole nitrogen source. Concentration of carbon sources are indicated
